# Something Else Going On? Diagnostic Uncertainty in Children with Chronic Pain and Their Parents

**DOI:** 10.3390/children7100165

**Published:** 2020-10-04

**Authors:** Vivek Tanna, Lauren C. Heathcote, Marissa S. Heirich, Gillian Rush, Alexandra Neville, Melanie Noel, Joshua W. Pate, Laura E. Simons

**Affiliations:** 1 Department of Anesthesiology, Perioperative, and Pain Medicine, Stanford University School of Medicine, Stanford, CA 94305, USA; vtanna@stanford.edu (V.T.); lcheath@stanford.edu (L.C.H.); mheirich@stanford.edu (M.S.H.); gqrush@stanford.edu (G.R.); 2 Department of Psychology, University of Calgary, Calgary, AB T2N 1N4, Canada; alexandra.neville@ucalgary.ca (A.N.); melanie.noel@ucalgary.ca (M.N.); 3 Alberta Children’s Hospital Research Institute, University of Calgary, Calgary, AB T2N 4N1, Canada; 4 Hotchkiss Brain Institute, University of Calgary, Calgary, AB T2N 4N1, Canada; 5 Graduate School of Health, University of Technology Sydney, Chippendale, NSW 2008, Australia; joshua.pate@uts.edu.au

**Keywords:** diagnostic uncertainty, pain, chronic pain, pediatric, child, parent, fear-avoidance, pain acceptance

## Abstract

Diagnostic uncertainty, the perceived lack of an accurate explanation of the patient’s health problem, remains relatively unstudied in children. This study examined the prevalence, familial concordance, and correlates of diagnostic uncertainty in children and their parents presenting to a multidisciplinary pain clinic in the United States. One hundred and twenty-six parents and 91 of their children (*M*_age_ = 13.93 years, range = 8–18 years) completed a brief three-item measure of diagnostic uncertainty, as well as measures of pain-related distress and functioning. Forty-eight percent of children and 37% of parents believed something else was going on with the child’s pain that doctors had not found out about yet. Across the three items, 66%–77% of children and their parents agreed in their endorsement of diagnostic uncertainty. Parents who believed that something else was going on with their child’s pain had children with higher avoidance of pain-related activities (*F* = 5.601, *p* = 0.020) and lower pain willingness (*F* = 4.782, *p* = 0.032). Neither parent nor child diagnostic uncertainty was significantly related to the child’s pain-related functioning. Diagnostic uncertainty, particularly in parents, is relevant in the experience of pediatric chronic pain and warrants further investigation as both a risk factor and therapeutic target.

## 1. Introduction

Chronic pain is common among children and adolescents (hereafter referred to as children), with median prevalence rates ranging from 11%–38% [1], and with 36% of these children seeking specialty care for their pain [2]. While chronic pain can result from an initial injury or illness, one third of children seeking care for chronic pain report no precipitating event preceding the onset of their pain [3]; thus, the apparent cause of the child’s pain can be elusive. When persistent pain cannot be explained by underlying pathophysiology, a child seeking care for their pain will often receive a diagnosis such as “chronic pain”, “amplified musculoskeletal pain disorder”, or “pain-associated disability syndrome”, which may be perceived as catch-all or default diagnostic labels [4]. Consequently, it is likely that some children with chronic pain, and their parents, will experience uncertainty around the child’s pain diagnosis, pathophysiology, and prognosis [5].

Diagnostic uncertainty has been defined as the clinician’s “subjective perception of an inability to provide an accurate explanation of the patient’s health problem” [6]. While this definition centers around the clinician, diagnostic uncertainty can also refer to patients’ perceptions that such an explanation is missing, or that the one given is inadequate for explaining their pain [7]. A complex phenomenon, diagnostic uncertainty can involve uncertainty around the specific diagnostic label, or the broader explanation provided for one’s symptom experience, or can pertain to the general feeling that something has been missed by clinicians. Evidently, the patient and family’s experience of diagnostic uncertainty heavily interacts with their communication with the healthcare provider as well as their interactions with the broader healthcare system [6,8]. Among adults, diagnostic uncertainty is emerging as an important factor associated with negative pain-related outcomes [9,10,11]. Adults with chronic low back pain with diagnostic uncertainty have higher levels of depression and disability compared to those without diagnostic uncertainty, in addition to a recall bias for illness-related stimuli [10]. They also report greater pain-related distress and further treatment-seeking [9], indicating that diagnostic uncertainty may be relevant for understanding healthcare use associated with chronic pain.

In pediatric chronic pain, the nature, prevalence, and impact of diagnostic uncertainty has been largely overlooked, except for a few studies. Although not examining diagnostic uncertainty per se, an initial qualitative study found that parents often reported a struggle for coherence around their child’s chronic pain, with a clear diagnosis seen as a source of relief and validation [12]. In a more recent qualitative study, youth with chronic pain and their parents reported experiencing diagnostic uncertainty, which was tied to their trust in the medical system and the fear that something more serious had been missed by doctors [5]. Moreover, even after some children had received a diagnosis and treatment for their pain, children and parents often reported a continued search for an alternate diagnosis that would provide a sufficient explanation for the extent of the child’s pain [5]. Taken together, these findings suggest that the critical (and perhaps most impactful) facet of diagnostic uncertainty may revolve around the feeling that something has been missed in the diagnostic process, as has been found in adult samples.

In the first quantitative study in pediatric chronic pain, Neville and colleagues found that nearly a third of children and parents believed that something else was going on with the child’s pain that doctors had not yet found [4]. Moreover, 70% of children and parents were in agreement regarding their experience of diagnostic uncertainty, indicating moderate-to-high concordance within the family unit [4]. Children who reported diagnostic uncertainty also reported higher catastrophizing about their pain and marginally higher pain-related fear-avoidance, parents who reported diagnostic uncertainty had children who reported greater pain intensity, greater pain interference, lower health-related quality of life, and marginally higher pain-related fear-avoidance [4]. Thus, preliminary data indicate that child and parent diagnostic uncertainty is prevalent and potentially impactful in pediatric pain populations. Given that the aforementioned quantitative study was the first of its kind, from a single institution in Canada, and included a group of children with predominantly headache, these findings require replication in independent, geographically distinct samples with other chronic pain conditions.

In addition to replication, there is a need for extension. In particular, the association between child/parent diagnostic uncertainty and the child’s functional disability, a primary outcome in pediatric pain interventions, has not been studied. Moreover, children who think that something has been missed by the doctors may feel the need to continue searching for answers and thus may feel less accepting of their pain. In addition, while parent diagnostic uncertainty may influence the child’s response to their pain, it is also important to consider how parent diagnostic uncertainty influences the parents’ own response to and cognitions about their child’s pain. A large body of research has shown that parent cognitions, particularly catastrophizing about the child’s pain and resultant protective behaviors, are associated with poor child functioning [13,14]. Of particular relevance to diagnostic uncertainty is a study that found that parents who told a distress (versus resilience) narrative, characterized by more negative affect and an unresolved orientation toward the child’s diagnosis of chronic pain, reported higher levels of catastrophizing about their child’s pain (helplessness subscale) [15]. Relatedly, parents’ willingness to experience distress in the presence of their child’s pain in the service of valued behavior, termed ‘psychological flexibility’, is linked with both parent protective behavior and child functioning [16,17] and may be influenced by parents’ diagnostic uncertainty. Particularly, parents who feel confident in the accuracy of their child’s diagnosis may be more willing to tolerate the distress associated with their child’s pain. Taken together, these studies indicate that parent diagnostic uncertainty about their child’s pain may influence parent distress and psychological flexibility regarding their child’s pain.

In this study, we aimed to (1) replicate existing findings on the prevalence and concordance of child and parent diagnostic uncertainty in a US-based clinical sample of youth with varied pain locations, (2) replicate existing findings on the association between child and parent diagnostic uncertainty with child pain-related distress (catastrophizing, fear-avoidance), and (3) extend existing findings by examining associations between child and parent diagnostic uncertainty with child functional disability and pain acceptance, and between parent diagnostic uncertainty with parents’ catastrophizing and psychological flexibility regarding their child’s pain.

## 2. Materials and Methods

Children (aged 8 to 18 years) and their parents were recruited during the child’s initial evaluation at a multidisciplinary pain clinic at Stanford Children’s Health. Exclusion criteria for children included cognitive impairment or developmental delay (screened via medical record review), a lack of English reading/writing proficiency, or a cancer diagnosis. Children and parents provided informed assent and consent, respectively, and all measures were completed through research electronic data capture (REDCap). Children and parents also completed measures relating to the validation of a pain-related knowledge measure that was unrelated to the aims of this study and will be reported elsewhere. All study procedures were approved by the site institutional review board (IRB). All subjects gave their informed consent for inclusion before they participated in the study. All study procedures were approved by the Stanford University Institutional Review Board (IRB-#46499).

Families were approached by a clinical research coordinator to begin study measures during a standard break in the child’s clinical evaluations, during which the multidisciplinary clinical team met to discuss the treatment plan. In addition, due to the time constraint of completing measures in the clinic, for some participants (children *n* = 10; parents *n* = 19), fear of pain and pain catastrophizing data were extracted from the Collaborative Health Outcomes Information Registry (CHOIR), a battery of measures completed by families prior to each pain clinic visit with the intention of tracking patient outcomes over time. All participants for whom CHOIR data were used completed the study survey and the CHOIR measures within 9 days of one another (*M* = 1.6 days).

Within measures, mean imputation was performed where participants completed at least 80% of the items for a variable. In total, imputation was performed with 0.26% of data, or 16 total items among 7 children and 8 parents that were distributed across all variables. Due to the very low missing data rate and the fact that these were distributed across all variables, we concluded that any resulting bias was negligible [18].

### 2.1. Measures

*Demographic and pain variables.* Children reported their gender and the location(s) of their pain. Parents reported their gender and education level, as well as their child’s race. Parents also reported the start date of their child’s pain, and child pain duration was calculated as the time between the pain start date and the survey date.

*Diagnostic Uncertainty.* Diagnostic uncertainty was assessed with a brief, empirically derived measure of three yes/no items, with child and parent versions [4]. For each item, there were follow-up questions if the respondent answered “Yes”. These items were developed for adults with lower back pain [9] and subsequently adapted for any pain condition in a pediatric context [7]. In accordance with previous studies in adults and youth [4,7,10,11], item 3, which assessed the extent to which parents and children believed that something had been missed with respect to their child’s diagnosis (i.e., “something else going on”), was used to classify respondents into uncertain/certain groups to examine associations with outcomes. See Table 1 for a summary of the item content and branching logic.

#### 2.1.1. Child Measures

*Pain Intensity.* Typical child pain intensity (“typical or usual level of pain”) was assessed using an 11-point numerical rating scale from 0 (no pain) to 10 (worst pain possible) [19].

*Pain-Related Fear-Avoidance.* Child pain-related fear-avoidance was assessed using the Fear of Pain Questionnaire for Children-Short Form (FOPQC-SF) [20]. The measure consists of 10 items scored on a 5-point Likert scale, ranging from 0 for “Strongly Disagree” to 4 for “Strongly Agree”. In addition to the total score, the measure includes two subscales: fear of pain (4 items) and activity avoidance (6 items). Items were summed to calculate the total score and subscales, with higher scores indicating greater fear-avoidance. Item internal consistency for this measure within this sample was good for the total score (α = 0.83) and ranged from acceptable to good for the subscales (fear of pain α = 0.73, activity avoidance: α = 0.81).

*Pain Catastrophizing.* Child pain catastrophizing was assessed with the Pain Catastrophizing Scale-Child Version (PCS-C) [21]. The measure consists of 13 items scored on a 5-point Likert scale, ranging from 0 for “Strongly Disagree” to 4 for “Strongly Agree”. Items were summed, with higher scores indicating greater pain catastrophizing. Internal consistency for this measure within this sample was excellent (α = 0.91).

*Functional Disability.* Child functional impairment was assessed with the Functional Disability Inventory (FDI) [22]. The measure consists of 15 items scored on a 5-point Likert scale, ranging from 0 for “Strongly Disagree” to 4 for “Strongly Agree”. Items were summed, with higher scores indicating greater functional impairment due to pain. Internal consistency for this measure within this sample was good (α = 0.89).

*Chronic Pain Acceptance.* Child chronic pain acceptance was assessed with the Chronic Pain Acceptance Questionnaire, 8-item short form version for children (CPAQ-8) [23]. Items were rated on a 5-point Likert scale, ranging from 0 for “Strongly Disagree” to 4 for “Strongly Agree”. In addition to the total score, the measure includes two subscales: activity engagement (4 items) and pain willingness (4 items). Items were reversed scored where indicated and summed to calculate total and subscale scores [23], with higher scores indicating greater acceptance of pain. Internal consistency for the total and subscales within this sample was acceptable (total α = 0.719, activity engagement α = 0.787; pain willingness α = 0.732).

#### 2.1.2. Parent Measures

*Pain Catastrophizing.* Parent pain catastrophizing was assessed with the Parent Pain Catastrophizing Scale (P-PCS) [24]. The measure consists of 13 items scored on a 5-point Likert scale, ranging from 0 for “Strongly Disagree” to 4 for “Strongly Agree”. Items were summed, with higher scores indicating greater catastrophizing about their child’s pain. Internal consistency for this measure within this sample was excellent (α = 0.92).

*Parent Psychological Flexibility.* Parent psychological flexibility regarding their child’s pain was assessed using the Parent Psychological Flexibility Questionnaire, 10-item version (PPFQ-10) [16,17]. Items are rated on a 7-point Likert scale, from 0 for “Never” to 6 for “Always”. In addition to the total score, the measure includes three subscales: value based action (3 items), emotional acceptance (4 items), and pain willingness (3 items). Items were reverse scored where indicated [16,17] and summed to calculate the total score and three subscales, with higher scores indicating greater psychological flexibility. Internal consistency for the total score within this sample was good (α = 0.83) and ranged from acceptable to good for subscales (value based action α = 0.75, emotional acceptance α = 0.88, pain willingness α = 0.72).

### 2.2. Analyses

Descriptive statistics were used to assess the prevalence of diagnostic uncertainty in children and parents. To assess differences between items as well as concordance between children and parents, chi square analyses were performed between the three diagnostic uncertainty items within as well as between child and parent reports, respectively. Chi square and t-test analyses were performed to examine differences in diagnostic uncertainty according to child gender and age. Analyses of covariance (ANCOVA), with child age and gender as covariates, were performed to assess the relation between diagnostic uncertainty (with those who answered “Yes” to item 3 classified as uncertain) and the other child and parent self-report measures of pain-related distress and functioning. Given the hypothesis-generating nature of this study, we did not apply a stringent multiple test correction for significance. All analyses were performed in SPSS version 26.

## 3. Results

In total, 129 children and their parents took part in the larger survey study. Children and their parents were predominantly female, and most children reported multi-site pain; sample demographic and pain characteristics are summarized in Table 2. Nearly all parents (*N* = 126) completed the diagnostic uncertainty measure, while 91 children completed the measure. In addition, two parents did not complete the diagnostic uncertainty measure but were included because their child did, and one parent only completed item one from the diagnostic uncertainty measure. To maximize sample size and thus power, we retained the maximum possible data across each analysis and thus we report sample sizes for each analysis.

Since families were mostly recruited during a break in clinical evaluation, by comparing survey timestamps with evaluation start times, we could estimate whether the diagnostic uncertainty measure was completed before or after the conclusion of the evaluation. We estimated that all children completed the diagnostic uncertainty measure after evaluation, while only 64.8% of parents completed the diagnostic measure after evaluation. This difference is explained by the parent survey being presented first on a shared device. Of note, parent diagnostic uncertainty was unrelated to whether the measure was completed before vs. after the conclusion of the clinical evaluation (item one: *X*^2^ = 1.475, *p* = 0.153; item two: *X*^2^ = 0.383, *p* = 0.333; item three: *X*^2^ = 0.317, *p* = 0.354).

### 3.1. Prevalence and Concordance of Diagnostic Uncertainty in Parents and Children

**Figure 1:**
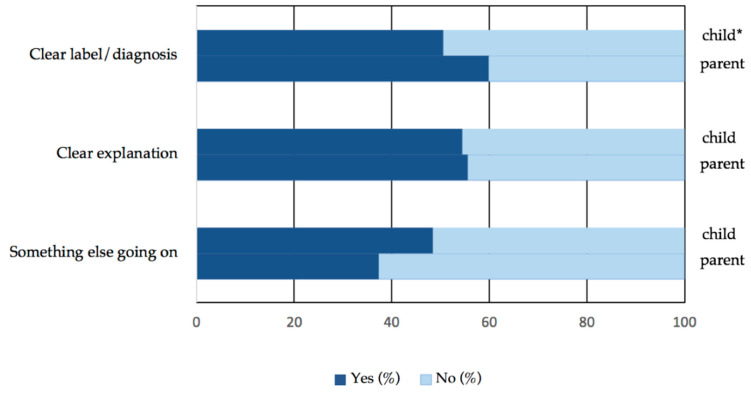
Diagnostic uncertainty quantitative item responses. * Item 1 (clear label/diagnosis): child *n* = 91, parent *n* = 127; item 2 (clear explanation) child *n* = 90, parent *n* = 126; item 3 (something else going on) child *n* = 91, parent *n* = 126.

Descriptive statistics on diagnostic uncertainty quantitative item responses are provided in Figure 1, and diagnostic uncertainty qualitative item responses in Table 3. Across the first two diagnostic uncertainty items (receiving a clear label/diagnosis and explanation), there was a relatively even divide for both parent and child responses. All parents (100%) who reported that they had received a clear label/diagnosis for their child’s pain agreed with that diagnosis, and nearly all parents and children who reported that they had been given a clear explanation agreed with that explanation (parents 98.6%, children 97.9%). For item three, 48.4% of children and 37.3% of parents believed something else was going on that the doctors had not found out about yet. As detailed in Table 3, there was a range of responses to diagnoses and to what else was going on that doctors had missed. Children and parents often admitted that they did not know what else was going on, and often reported seeking a “source” or “underlying cause” for the child’s pain that doctors had not found out about yet.

Reports of receiving a clear label/diagnosis and receiving a clear explanation for the chronic pain were highly overlapping, with 88.1% of children (X^2^ = 40.1, *p* < 0.001) and 80% of parents (X^2^ = 44.8, *p* < 0.001) who reported receiving a clear label/diagnosis also reporting receiving a clear explanation for the child’s chronic pain. For parents, those who reported receiving a clear diagnosis (item one) and those who reported receiving a clear explanation for chronic pain (item two) were both less likely to report that something else was going on that doctors had not found out about yet (item one: X^2^ = 9.9, *p* = 0.002; item two: X^2^ = 28.8, *p* < 0.001). For children, only those who reported receiving a clear explanation for chronic pain (item two), but those who reported receiving a clear diagnosis (item one), were less likely to report that something else was going on that doctors had not found out about yet (item one: X^2^ = 3.2, *p* = 0.058; item two: X^2^ = 3.5, *p* = 0.049). Yet, of those who reported that they had received a clear label/diagnosis, 39.1% of children and 26.3% of parents still believed that something else was going on that doctors had not found out about yet.

For concordance analyses, the diagnostic uncertainty measure was complete for 88 total parent-child dyads (89 dyads for item one). For all three diagnostic uncertainty items, there were moderate-to-high levels of agreement between children and parents (item one: 76.4% concordance, X^2^ = 24.89, *p* < 0.001; item two: 71.6% concordance, X^2^ = 16.50, *p* < 0.001; item three: 65.9% concordance, X^2^ = 8.94, *p* = 0.005).

Diagnostic uncertainty did not differ significantly by child gender, but children who reported diagnostic uncertainty were, on average, one year older (14.4 vs. 13.4 years, F = 0.596, *p* = 0.045). The perception of children or parents that something else was going on that doctors had not found out about yet was not significantly related to child pain duration (child t = 0.757, *p* = 0.452; parent t = 1.225, *p* = 0.223).

### 3.2. Diagnostic Uncertainly in Relation to Child Pain-Related Fear-Avoidance and Catastrophizing

Older children had higher pain-related fear-avoidance total scores (*r* = 0.217, *p* = 0.046) and activity avoidance subscale scores (*r* = 0.236, *p* = 0.030) than younger children. No measures differed significantly by child gender, but analyses of covariances were still performed with child age and gender as covariates to replicate methods used in the quantitative study by Neville et al. [4].

Full ANCOVA results of child and parent pain-related distress and behavior by diagnostic uncertainty (item three), controlling for child age and gender, are summarized in Table 4. In summary, parents who believed that something else was going on with their child’s pain (i.e., item three) had children with higher fear of pain, including the activity avoidance subscale. In contrast to previous findings, we did not find that child diagnostic uncertainty was significantly associated with any child-reported outcomes.

### 3.3. Diagnostic Uncertainty in Relation to New Child and Parent Factors

Parent or child diagnostic uncertainty was not significantly related to child pain acceptance or pain-related functional impairment, and parent diagnostic uncertainty was not significantly related to parent pain catastrophizing. In contrast to expectations, parents who believed that something else was going on with their child’s pain reported higher pain willingness (subscale of the PPFQ-10).

## 4. Discussion

This is the second quantitative study to examine the prevalence and correlates of diagnostic uncertainty among children (10–18 years) with chronic pain and their parents. Our first aim was to replicate findings from Neville and colleagues [4] on the prevalence and concordance of child and parent diagnostic uncertainty, using a US-based sample of youth with varied pain locations. We found almost half of children (48%) and over one-third of parents (37%) reported diagnostic uncertainty, which we primarily operationalized as the perception that something else was going on with the child’s condition that had been missed by clinicians [7,9,10,11]. Nearly all parents or children who reported that they had been given a clear label/diagnosis or explanation for the child’s chronic pain agreed with that label/diagnosis or explanation, consistent with past findings in adults [10] and children [4]. Yet, approximately one-third of children and parents who reported that they had been given a clear label/diagnosis for the child’s pain still believed that something else was going on with the child’s pain, suggesting that diagnostic uncertainty may relate to a perceived inadequacy of the diagnosis even when that diagnosis is accepted by the child or parent. Qualitative responses by both children and parents to item three (“What else do you think is going on?”) commonly suggested that the “source” or “underlying cause” of the child’s pain had not yet been identified. These findings align with extant qualitative work showing that some families seek not only individual diagnoses but also sufficient and comprehensive explanations for the entirety of the child’s chronic pain [5]. Our findings on the prevalence of diagnostic uncertainty are also consistent with the small number of extant quantitative studies, which have revealed diagnostic uncertainty in a substantial minority of adults with chronic low back pain [9,10,11] and youth with chronic (primarily headache) pain [4]. Yet, it is notable that in the current sample we found slightly higher rates of diagnostic uncertainty than reported in the previous child study [4]. These differences may be driven by geographical location (e.g., U.S. versus Canada) or by the fact that the majority of children in our sample reported multisite pain. Multi-site pain has previously been associated with poorer emotional outcomes and greater functional impairment [25]. Differences in the experience of diagnostic uncertainty across chronic pain diagnoses and sites remains a critical question within this emerging research area.

Up to three-quarters of children and their parents agreed in their endorsement of diagnostic uncertainty across the three items, indicating a moderate-to-high level of agreement within the family unit and consistent with findings by Neville et al. [4]. Still, in both the study by Neville and colleagues [4] and the current study, around one-third of the parent-child dyads were not concordant in their experience of diagnostic uncertainty. Relatedly, the qualitative study by Neville and colleagues found that a shift in focus from the search for a diagnosis to function did not always occur in unison for the parent-child dyad [5]. More broadly, previous research in pediatric chronic pain populations has found that parents and their children are highly concordant in their levels of pain catastrophizing, but when they diverge, it can be problematic. For example, adolescents who reported high pain catastrophizing while their parents reported low pain catastrophizing experience higher levels of depressive symptoms and functional disability [26]. On the flipside, when parent-child dyads agree on the same treatment goal in an internet-delivered chronic pain self-management program, children report lower pain intensity post-treatment and at follow-up [27]. Taken together, these findings indicate that parent-child concordance in their core beliefs about and goals for the child’s pain experience, including diagnostic certainty, may positively influence the child’s symptoms and functioning. Further work in larger samples will be needed to examine the role of concordance in diagnostic uncertainty. In particular, it will be interesting to assess whether parent-child concordance in diagnostic uncertainty is only predictive of child outcomes when the parent and child both experience a lack of diagnostic uncertainty, which we were not powered to examine.

We also aimed to replicate and extend existing findings on associations between child/parent diagnostic uncertainty and child pain outcomes, particularly the child’s pain-related distress (replication), acceptance (extension), and functioning (extension). We found that, when controlling for the child’s age and gender, parents with diagnostic uncertainty had children with significantly higher pain-related fear-avoidance, including the child’s avoidance of activities due to pain. We did not find that any parent or child measures of pain-related distress, acceptance, or functioning differed significantly between child certain and uncertain groups, although this may be due to limited power. Interestingly, we did not replicate the findings from Neville and colleagues [4] that found that children whose parents experienced diagnostic uncertainty reported greater pain intensity or catastrophizing. However, overall, our findings are consistent with Neville and colleagues’ findings that diagnostic uncertainty among parents appears relevant to child outcomes.

Finally, this was the first study to examine associations of parent diagnostic uncertainty with parent catastrophizing and psychological flexibility regarding their child’s pain. In contrast to expectations, we found that parent diagnostic uncertainty was not linked with parent pain catastrophizing but was linked with greater parent pain willingness (a subscale of the Parent Psychological Flexibility Questionnaire; PPFQ-10). That is, parents who reported diagnostic uncertainty also reported higher willingness to experience distress related to their child’s pain, in the service of valued behavior. This finding was not significant for the PPFQ-10 total score, and only just reached significance for the pain willingness subscale (*p* = 0.049) and thus should be interpreted with caution. However, if replicated, one possible explanation for this finding is that some parents who endorse a complete diagnosis for their child (i.e., who do not believe that anything has been missed) may still believe that their child’s pain condition is severe and thus that their child’s body is fragile. Indeed, close examination of the items for the PPFQ-10 pain willingness subscale indicate that they reflect the parents’ promotion of their child’s engagement with (potentially painful) activities. We may thus expect a moderating effect of parent beliefs about the severity of their child’s condition, such that parents with diagnostic uncertainty only report higher pain willingness if they do not believe that their child’s condition warrants high protective behaviors. The type of diagnosis that the child received, and the perceived severity associated with this diagnosis, likely also plays a moderating role. These hypotheses remain to be tested in a larger sample with sufficient power for moderation analyses.

This study has limitations, pointing towards directions for future research. First, we were underpowered to detect small and small-to-moderate effects for some measures; thus, our inability to replicate some of the findings by Neville and colleagues [4] may be due to a lack of power. Second, the cross-sectional design prohibited understanding of how diagnostic uncertainty fluctuates over time. Further work is particularly needed to examine how diagnostic uncertainty may change in response to pain treatment or education. Third, only total scores were available for the child Pain Catastrophizing Scale when pulled from the CHOIR registry, precluding analyses with subscales for this measures, although Pielech and colleagues have argued that pain catastrophizing is a unitary construct in children [28].

Another potential limitation of this study was the varied timing of when parents completed the diagnostic uncertainty measure with respect to the child’s pain clinic evaluations and treatment recommendations. However, we found that parent diagnostic uncertainty (item three) did not differ between groups that completed the measure before vs. after clinical evaluation. Interestingly, parent reports of receiving a clear label/diagnosis (item one) or a clear explanation for their child’s chronic pain (item two) also did not differ between these groups. These findings can be considered in the context of past studies that have found that diagnostic uncertainty appears unrelated to what patients think they were told about their pain and condition, but rather their prior beliefs and expectations in combination with communication with clinicians [5,10]. Given that all families referred to the specialty clinic have a history of evaluations by previous specialists, our data likely pertain to their uncertainty around several clinical interactions and do not speak per se to youth who have completed a multidisciplinary treatment program (which involves extensive pain education). Still, given that we could only compare distinct groups of parents, the potential impact of clinical communication on diagnostic uncertainty for any particular individual could not be examined.

Relatedly, our findings have relevance for parent-child communication with health care providers in the context of chronic pain [6,7]. The present study did not directly examine the family-provider relationship, but some qualitative responses to item three suggested that children and parents felt that clinicians did not appear to fully understand the child’s condition. Similarly, in the qualitative study by Neville and colleagues, diagnostic uncertainty among parents and youth appeared closely related to mistrust in the medical system, and a sense of not being listened to and validated by clinicians [5]. Children and parents in that study also reported that the perception that clinicians did not understand their child’s condition undermined their trust in the clinician and treatment buy-in [5]. Poor trust in clinicians may be a barrier to treatment adherence, as high parent-reported patient satisfaction and positive expectations of treatment results have been associated with better treatment adherence in pediatric chronic pain [29]. From the perspective of health care providers, one thematic analysis found that pediatric providers also experience diagnostic uncertainty, but how much they communicate their own diagnostic uncertainty likely depends on parent characteristics and the strength of the parent-provider relationship [30]. In addition, some qualitative diagnostic uncertainty responses in the present study indicated an intent to see additional providers. This, in combination with the previous qualitative study in which diagnostic uncertainty led children and their parents to a search for a satisfying diagnosis across multiple providers [5], suggests that the number of providers seen may be relevant to examine in relation to diagnostic uncertainty. Further work is needed to characterize provider communication in relation to diagnostic uncertainty, and how diagnoses or pain education might be conveyed to reduce the family’s diagnostic uncertainty.

The diagnostic uncertainty tool is short and accessible, allowing for high utility in a clinical or research setting. It also allows for the identification of diagnostic uncertainty even among those who report having received a clear diagnosis for the child’s pain, and across a variety of pain diagnoses. However, it is possible that not only diagnostic uncertainty, but also intolerance of uncertainty is of relevance for child outcomes. A recent longitudinal study by Neville and colleagues suggested that illness uncertainty in both children and their parents contributes to increases in child pain interference over time, via increased pain catastrophizing, parent protectiveness, and child fear of pain [31]. Thus, future work should consider not only diagnostic uncertainty but also intolerance of uncertainty as a risk factor for youth outcomes and potential target for intervention [31]. While useful, the diagnostic uncertainty tool should be supplemented with the child’s and parent’s own narrative about their pain and diagnostic journey.

## 5. Conclusions

Children seeking treatment for chronic pain, and their parents, commonly report diagnostic uncertainty with respect to the child’s pain condition. Parents and their children had high levels of agreement in their level of diagnostic uncertainty, and parent diagnostic uncertainty in particular was associated with higher child pain-related fear-avoidance (particularly activity avoidance). Further research is needed to better define the impact of communication between the family and health care provider on the family’s experience (and remittance) of diagnostic uncertainty, and how diagnostic uncertainty may change over time, within the context of pediatric chronic pain. 

## Figures and Tables

**Table 1:** Three-item measure of perceived diagnostic uncertainty.

Item		Answer
1. I have been given a clear label/diagnosis for my (my child’s) chronic pain.		Yes/No
	a. What label/diagnosis have you been given?	(open)
	b. Generally speaking, I agree with this label/diagnosis.*	Yes/No
2. I have been given a clear explanation about why I have (my child has) chronic pain.		Yes/No
	a. Generally speaking, I agree with this explanation.	Yes/No
3. I think there is something else happening with my (my child’s) chronic pain which the doctors have not found out about yet.		Yes/No
	a. What do you think is going on that the doctors have not found out about yet?	(open)

* Due to a programming error, this question was omitted from the child version.

**Table 2:** Demographic and pain characteristics.

Sample Characteristics		
Child age (*M* years, *SD*)		13.93 (2.46)
Child gender (% female)		68.3
Parent gender (% female)		93.7
Child race (%)		
	White	41.9
	Black	2.3
	Asian	5.4
	Hispanic	14.7
Parent education (% of *n* = 72)		
	High school or less	25.0
	Associate	23.6
	Undergraduate	25.0
	Postgraduate	26.4
Child pain location (% of *n* = 122)		
	Multiple locations	59.0
	Head	42.6
	Leg or foot	41.8
	Back	38.5
	Abdomen or stomach	34.4
	Shoulder or neck	32.8
	Arm or hand	25.4
	Chest	20.5
	Face or jaw	13.1
Average pain intensity (*M*, *SD*)		5.75 (1.96)
Pain duration in months (*Mdn*, range)		32.5 (1.2–212.9)

**Table 3:** Diagnostic uncertainty qualitative item responses.

Question	Responses	
**What label/diagnosis have you been given?**	Ehlers Danlos Syndrome, Complex Regional Pain Syndrome, Chronic Migraine, Fibromyalgia, hypermobility syndrome, Central Sensitization Syndrome, Connective Tissue Disorder, Postural Orthostatic Tachycardia Syndrome, carpal tunnel, Eosinophilic Esophagitis and IBS, Amplified Musculoskeletal Pain, Occipital Neuralgia, Epidermolysis Bullosa, back surgery pain, Meniscus tear & tendonitis in shoulder, Post Concussive Syndrome, juvenile ideopathic arthritis, injured nerve, injured rib cage muscles, Cerebrospinal fluid leak, depression migraines, neck pain from concussion, hypermobility syndrome, ankylosing spondylitis, benign joint hypermobility syndrome, neuropathic pain caused by trauma and degenerative back disease, gastritis, Chronic Recurrent Multifocal Osteomyelitis, Dysautonomia, Tendonitis, Reactive Airway Disease and Interstitial Cystitis, Erythromelalgia, Schwannomatosis, Chiari Malformation, rectal prolapse, Endometriosis, pancreatitis	
	Parents	Children
**What do you think is going on that doctors have not found out about yet?**	“Why the pain medication and treatments aren’t working” “Migraines are a guessing game.” “I have no idea. I am a graphic designer and not in the healthcare industry.” “Genetic conditions” “Still struggling to understand what kind of dysbiosis exists in her gut microbiome and how to improve it” “Why it’s happening and progression” “I don’t know, but nothing has helped” “Underlying cause” “sinus issues too” “I don’t know. That is why we are exploring many options” “I don’t know - but there is something we are all missing.” “His neurotransmitter levels are off with serotonin and dopamine levels excessively high.” “Cause for the pain” “?” “Possible endometriosis” “I don’t know.” “Her daily fever is not being explained.” “A concrete diagnosis” “Why she has pain” “Extent of nerve damage” “I don’t know” “Doctors have mentioned she has some inconsistencies as far as CRPS goes but have not been provided further explanation or reasoning” “Source of pain” “We have the initial diagnosis of functional abdominal pain, but my understanding is that this is not really solidly understood, so we could be in for some unanswered questions and false starts.” “Clear diagnosis” “Some kind of metabolic/genetic disorder” “Not sure” “Something neurological” “is there something underlying that is causing all of these things?” “I’m not sure, which is why we are also going to see a geneticist and possibly a cardiologist.” “I think they are unsure of the cause” “Possible thermal burns to nerves”	“They have not seen anyone with chronic pain in the same location” “They don’t have a solution” “Eds. Chiari” “What my pain is and what the root cause is of all my symptoms” “why it’s happening and how long it will last” “I’m not sure, but I hope they find something that can be solved easily” “What’s causing it” “What is causing the pain and why it was triggered in the first place” “Not sure” “I don’t know.” “I’m not sure because I haven’t gotten told about what is wrong with me” “Exactly where the pain is coming from” “I don’t know but I hate my headaches” “Why it started and what exactly is causing it” “They have no idea what’s wrong” “Why my back hurts” “Honestly I think there’s probably a strong psychological aspect to my pain” “My body is kind of weird, and I don’t think any of my doctors have spent enough time with me to really understand how it works” “I don’t really know but it feels like I’m missing something” “I think they don’t understand how much pain I can be in” “What is the original cause of it and if there is a way to get rid of it” “Fatigue” “Don’t know just have a feeling they haven’t found something yet.” “Neurological issues” “Not sure” “I don’t know, but I think there is something causing all this pain” “The source and cause of my pain” “I’m not entirely sure, but a clear diagnosis has not been given.” “Electrical injuries are rare and aren’t studied upon by many doctors” “I just feel like there IS something physically wrong with the nerves in my neck, like they are fundamentally damaged” “The root cause and why the headaches even start in the first place”

**Table 4:** Analyses of covariance (ANCOVA) results by diagnostic uncertainty, controlling for child age and gender.

Variable		*N*	Certain (*M*, *SD*)	Uncertain (*M*, *SD*)	*F*	*df*	*p*
**Parent Diagnostic Uncertainty**							
Average Pain Intensity		109	5.63 (2.15)	6.07 (1.54)	1.529	1, 105	0.232
Child Pain Catastrophizing		82	22.70 (11.27)	25.40 (9.32)	1.446	1, 78	0.221
Child Functional Disability		107	20.43 (10.20)	24.36 (11.15)	2.639	1, 103	0.107
Child Fear of Pain		82	19.76 (8.09)	23.73 (4.94)	5.987	1, 78	**0.017**
	Fear of Pain	81	6.84 (3.89)	8.23 (2.30)	3.225	1, 77	0.076
	Activity Avoidance	82	12.83 (5.49)	15.50 (3.62)	5.601	1, 78	**0.020**
Child Pain Acceptance		86	16.64 (4.63)	15.34 (5.09)	1.518	1, 82	0.221
	Pain Willingness	86	6.90 (3.02)	5.71 (3.59)	3.014	1, 82	0.086
	Activity Engagement	86	9.84 (2.92)	9.58 (3.16)	0.105	1, 82	0.747
Parent Pain Catastrophizing		88	22.37 (9.89)	21.86 (10.09)	0.050	1, 84	0.824
Parent Psychological Flexibility		122	34.44 (8.81)	37.28 (8.55)	3.250	1, 118	0.074
	Value Based Action	121	11.27 (3.18)	11.62 (2.79)	0.503	1, 117	0.480
	Emotional Acceptance	121	15.39 (5.34)	16.63 (4.97)	1.768	1, 117	0.186
	Pain Willingness	121	7.71 (3.18)	8.98 (3.40)	3.967	1, 117	**0.049**
**Child Diagnostic Uncertainty**							
Average Pain Intensity		91	5.53 (1.99)	5.66 (1.84)	0.477	1, 87	0.492
Child Pain Catastrophizing		65	23.60 (10.79)	22.00 (9.05)	0.900	1, 61	0.347
Child Functional Disability		91	20.67 (10.03)	22.22 (11.23)	0.402	1, 87	0.528
Child Fear of Pain		65	21.12 (7.25)	20.73 (6.37)	0.730	1, 61	0.396
	Fear of Pain	64	7.74 (3.37)	6.77 (2.91)	2.624	1, 60	0.111
	Activity Avoidance	65	13.29 (5.15)	13.97 (4.46)	0.001	1, 61	0.970
Child Pain Acceptance		89	16.67 (4.16)	15.55 (5.37)	1.477	1, 85	0.228
	Pain Willingness	89	6.68 (2.65)	6.19 (3.83)	0.353	1, 85	0.554
	Activity Engagement	89	10.11 (2.71)	9.33 (3.21)	2.430	1, 85	0.123

Bold number means they have reached the threshold for significance.
